# Solvation Dynamics of CO_2_(g) by Monoethanolamine at the Gas–Liquid Interface: A Molecular Mechanics Approach

**DOI:** 10.3390/molecules22010008

**Published:** 2016-12-23

**Authors:** I-Shou Huang, Jia-Jen Li, Ming-Kang Tsai

**Affiliations:** Department of Chemistry, National Taiwan Normal University, Taipei 11677, Taiwan; FerrisWhiston@gmail.com (I-S.H.); sophy0956@gmail.com (J.-J.L.)

**Keywords:** CO_2_ capture, alcoholamine, molecular mechanics, interface, dynamics

## Abstract

A classical force field approach was used to characterize the solvation dynamics of high-density CO_2_(g) by monoethanolamine (MEA) at the air–liquid interface. Intra- and intermolecular CO_2_ and MEA potentials were parameterized according to the energetics calculated at the MP2 and BLYP-D2 levels of theory. The thermodynamic properties of CO_2_ and MEA, such as heat capacity and melting point, were consistently predicted using this classical potential. An approximate interfacial simulation for CO_2_(g)/MEA(l) was performed to monitor the depletion of the CO_2_(g) phase, which was influenced by amino and hydroxyl groups of MEA. There are more intramolecular hydrogen bond interactions notably identified in the interfacial simulation than the case of bulk MEA(l) simulation. The hydroxyl group of MEA was found to more actively approach CO_2_ and overpower the amino group to interact with CO_2_ at the air–liquid interface. With artificially reducing the dipole moment of the hydroxyl group, CO_2_–amino group interaction was enhanced and suppressed CO_2_(g) depletion. The hydroxyl group of MEA was concluded to play dual but contradictory roles for CO_2_ capture.

## 1. Introduction

Monoethanolamine (MEA) is extensively used in the electric power industry for directly scrubbing CO_2_ from the exhaust gas produced by fossil fuel combustion [1]. MEA nucleophilically attacks the partially positively charged carbon atom of CO_2_ with its amino groups, forming the zwitterionic (ZW) intermediate of [HOCH_2_CH_2_NH_3_^+^CO_2_^−^], thereby pulling CO_2_ from the exhaust gas. This ZW intermediate subsequently stabilizes itself by transferring a proton from its quaternary nitrogen through an intermolecular hydrogen bond (HB) network to other proton acceptors. Such a network can be formed by a combination of the amino or hydroxyl groups of MEA or with other additives that can form HBs. Water and other types of alcoholamines, such as diethanolamines (DEAs) and methyl-diethanolamines (MDEAs), are common additives in MEA solutions to improve overall CO_2_ scrubbing efficiency.

MEA is widely produced by controlling the stoichiometry of ethylene oxide reacting with aqueous ammonia. Secondary or tertiary amines can be generated by increasing the ethylene oxide:ammonia ratio. Several studies have performed experimental characterizations [2,3,4,5,6,7,8,9,10,11,12,13,14] and computational investigations [15,16,17] to elucidate and improve CO_2_-scrubbing reactivity by using various types of alcoholamines. The engineering of various functionalized ethylene oxides as the starting materials at the industrial scale to synthesize the designed alcoholamines can be challenging. Conway et al. measured the kinetic rate and equilibrium constants of CO_2_ (aq) with various sterically hindered alcoholamines and determined iso-butylamine, *n*-butylamine, and *n*-propylamine to be the main scrubbers for the CO_2_ binding step; none of the three amines contain hydroxyl groups [13]. A hydroxylated compound, 3-amino-1-propanol (MPA), was observed to be approximately 20% less efficient at binding CO_2_ (aq) compared with the aforementioned amines. Intuitively, elongating the alcohol groups, such as MPA in respect to and MEA, can improve CO_2_ binding affinity. Li and Tsai performed molecular dynamics simulations and stated that an appropriate length of the alkyl chain between the amino and hydroxyl groups can minimize the electronic cancellation effect from the two electron-withdrawing groups [18]. Adding water molecules to alcoholamines can facilitate the deprotonation process [19] and stabilize the ZW intermediates. However, water molecules can also potentially compete for the CO_2_ absorption sites by forming intermolecular HBs with the amino groups [18]. If the basicity of alcoholamines is adequately strong, water can be deprotonated to form OH^−^ and protonated alcoholamines, where OH^−^ is known to bind CO_2_ efficiently.

Considering the aforementioned observations, the hydroxyl groups of alcoholamines seem to play a trivial role for CO_2_ capture. However, because of the use of ethylene oxide as the starting material for synthesizing alcoholamines, the hydroxyl groups are inherently present in CO_2_ scrubbers. Compared with the CO_2_-binding characteristic of the amino groups, the hydroxyl groups can shuttle protons from the quaternary nitrogen of the ZW intermediates to the CO_2_^−^ moieties, as shown by ab initio molecular dynamic simulations of CO_2_ in neat MEA liquid [20]. However, this proton transfer capability of the hydroxyl groups is anticipated to be less effective compared with that of water molecules.

Several studies have performed computational modeling of the CO_2_ capture phenomena at the nanometer and nanosecond scales by using a classical force field approach [21]. Most of these studies primarily focused on predicting the CO_2_ transport property and fixation phenomena through physical absorption in various solutions, such as CO_2_, H_2_O, and a mixture of different types of alcoholamine derivatives. Jhon et al. predicted the nucleophilicities and accessibilities of MEA, 2-methylaminoethanol, and 2-amino-2-methyl-1-propanol, and emphasized that the predicted CO_2_-binding reactivity correlates with the accuracy of describing the steric effect of amines by the chosen force field [22]. Turgman-Cohen et al. included the ionic species that resulted from CO_2_ chemisorption in the model and qualitatively mimicked the thermogravimetric experiments [23]. Farmahini et al. simulated the separation of CO_2_ and CH_4_ mixtures in MDEA solution, which was accelerated piperazine [24]. Yu et al. developed a synergistic molecular dynamics model and reported that an optimal ternary mixture outperformed other quaternary or quintuple amine systems in the realm of CO_2_ diffusivity [25].

Extensive computational studies have focused on improving CO_2_-binding efficiency by using amino groups; however, the roles of the hydroxyl groups of alcoholamines have not been fully characterized. Notably, Du et al. measured the surface vibrational spectrum by using the sum frequency generation technique to address the hydrophobicity of the air–water interface and identified the spectroscopic fingerprint of the dangling hydroxyl groups [26]. Electrostatic interactions resulting from such dangling hydroxyl groups at the air–water interface favored the solvation of anionic ions, as previously reported [27,28,29,30,31,32,33]. Thus, the solvation phenomena of nonpolar CO_2_ molecules containing the quadruple moment at the air–alcoholamine interface inspired the present study. Pure CO_2_(g) can be considered a hydrophobic phase, whereas scrubber alcoholamines are highly hydrophilic because of the presence of amino and hydroxyl groups. Understanding the dynamic details of removing CO_2_ molecules from the hydrophobic environment to be subsequently solvated and absorbed by hydrophilic alcoholamines can provide the underlying microscopic characteristics of these organic scrubbers.

Therefore, we conducted all-atom molecular dynamic simulations to model CO_2_ solvation dynamics at the air–liquid interface. We started from the MM3 force field [34,35,36,37,38,39], a classical molecular mechanics force field for hydrocarbons, and modified the required parameters for CO_2_ and MEA molecules, then benchmarked the CO_2_ potential in the gas phase, MEA(l) potential in the solution phase, and the CO_2_–MEA absorption pathway compared with density functional theory (DFT) results. Finally, we monitored the dynamics of the CO_2_(g)/MEA(l) interface. Proton transfer from the ZW intermediates for the subsequent chemical absorption step was omitted in this study due to the complexity of parameterizing the reactive force fields. The present study has focused at the dominant physical absorption phenomena of CO_2_ molecules at the air–liquid interface.

## 2. Results and Discussion

### 2.1. Benchmark Using CO_2_ and (CO_2_)_2_ Models

**Figure 1:**
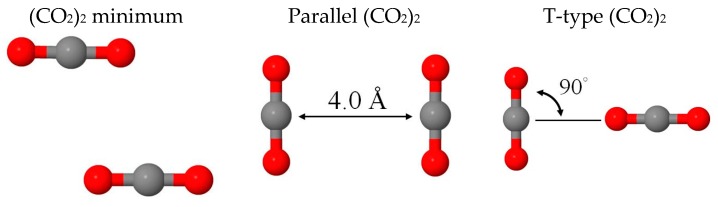
Schematic representation of the minimum, parallel and T-type (CO_2_)_2_ structures.

The intermolecular interactions between CO_2_ molecules of three selected characteristic structures—namely, the global minimum, parallel, and T-type (CO_2_)_2_ structures in Figure 1—were compared at the BLYP-D2, MP2, and molecular mechanics (MM) levels of theory in Table 1. Geometric constraints were imposed for optimizing the parallel and T-type structures. The intermolecular interactions (Eint) characterized at the MM level were compared using the binding energies (BE) of the BLYP-D2 or MP2 levels. For the global minimum structure of (CO_2_)_2_, Eint was predicted to be −1.23 kcal/mol versus −1.06 and −1.27 kcal/mol at the BLYP-D2 and MP2 levels, respectively. The parallel (CO_2_)_2_ structure with C–C constrained at 4.0 Å was the most repulsive one, where the MM level (0.16 kcal/mol) qualitatively reproduced the interaction, as predicted at the BLYP-D2 and MP2 levels (0.25 and −0.01 kcal/mol). T-type (CO_2_)_2_ was consistently predicted to interact less strongly than the global minimum at all three levels. The current nonpolarizable MM approach dominantly contributed through dipole–dipole (μμ) interactions for intermolecular CO_2_ interactions, but van der Waals (vdW) interactions primarily contributed to the BE of BLYP-D2 and MP2 levels. Using the functional forms of classical electrostatic and Lennard–Jones (LJ) potentials to distinguish the electrostatic and vdW contributions have been reported as challenging in the case of water model development [40,41,42,43]. The success of such distinction is mainly subject to the selected functional form for the classical representation of vdW interactions. Thus, further improvement of current CO_2_ potential is in progress and not included in the scope of this study. Nonetheless, the total intermolecular CO_2_ interaction calculated by MM still remained a reasonable agreement with the quantum mechanical (QM) results. Finally, vibrational frequencies of the CO_2_ monomer calculated at the MM level were consistent with those calculated at the BLYP-D2 and MP2 levels.

### 2.2. Phase Transition of (CO_2_)_13_

**Figure 2:**
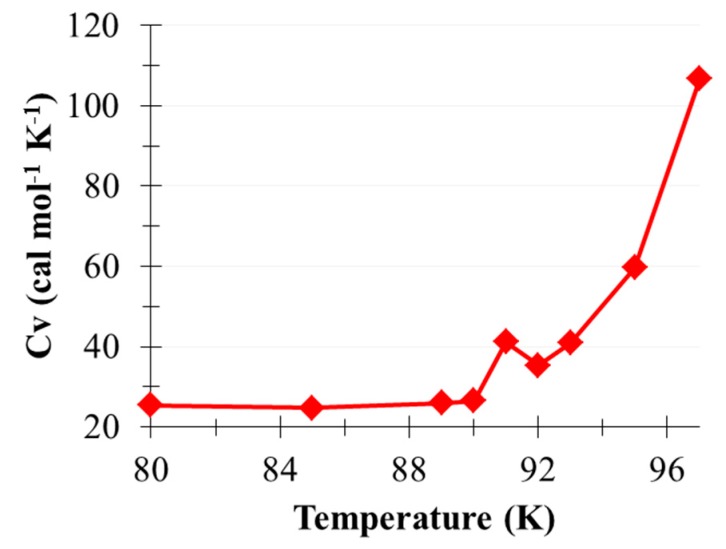
Heat capacity curve of (CO_2_)_13_ clusters predicted using molecular dynamic simulations.

The phase transition temperature of (CO_2_)_13_ clusters was characterized to benchmark the thermodynamic property prediction by using the current CO_2_ potential. Maillet et al. reported the melting transition of (CO_2_)_13_ at 95 K [44]. Liu and Jordan observed the solid–solid phase transition at approximately 90 K [45] by using a two-body model developed by Murthy et al. [46]. Murthy potential is a two-body potential using a rigid monomer, 5-point electrostatic sites, and atomic 6–12 Lennard–Jones terms. Comparably, the current CO_2_ model showed vibrational capability, with atomic 6–12 Lennard–Jones terms and bond-centered dipole–dipole (μμ) interactions. The calculated heat capacity curve from 88 K to 97 K of (CO_2_)_13_ clusters is shown in Figure 2. Each canonical ensemble (NVT) simulation was pre-equilibrated for 2.5 ns and sampled for 100 ns at a 0.5-fs time step. A solid–solid phase transition was identified at 91 K, followed by cluster evaporation beyond 97 K. The heat capacity was calculated using Equation 1.

(1) $$C_{\text{NVT}}\left(T\right)=\frac{\langle U^{2}\rangle -{\langle U\rangle }^{2}}{N_{A}\;k_{B}\;T^{2}}$$

where U is the sampled potential energy of (CO_2_)_13_, N_A_ is the Avogadro constant, k_B_ is the Boltzmann constant, 〈U^2^〉 and 〈U〉^2^ denote the mean of U^2^ and the square of the average U, respectively.

### 2.3. Benchmark of the MEA Potential

**Figure 3:**
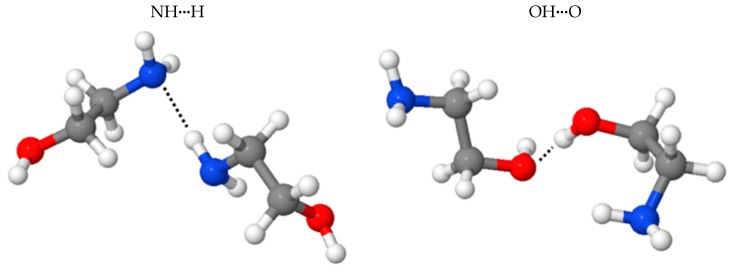
Constrained optimized geometries of (MEA)_2_ at the BLYP-D2/aug-cc-pVTZ level. MEA: monoethanolamine.

**Figure 4:**
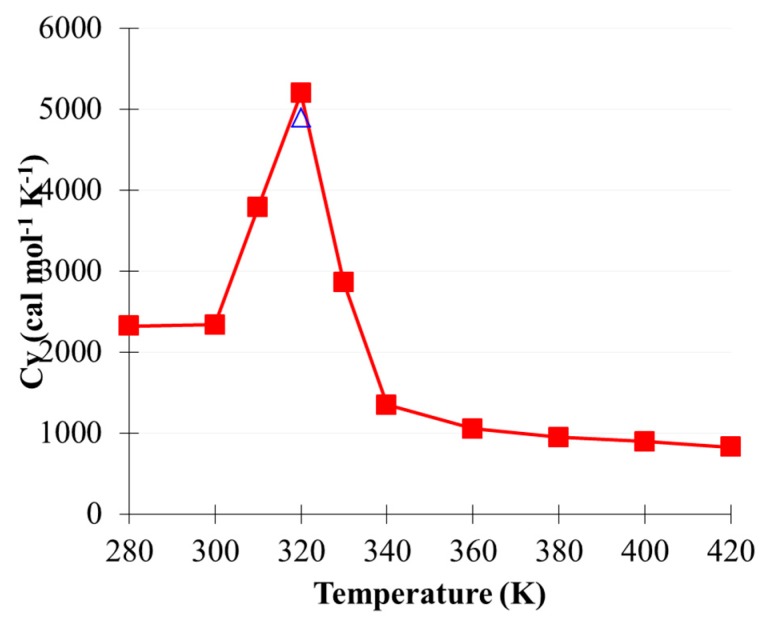
Heat capacity curve of the (MEA)_128_ model predicted using molecular dynamic simulation. The triangle denotes another independent 320 K simulation.

Three stable geometries are identified for the monomeric MEA at MP2/aug-cc-pVTZ with the polarizable continuum model (PCM) as shown in Figure S1. One of the *cis*-form MEA monomers containing an intramolecular hydrogen bond (intraHB) interaction between OH···NH_2_ is predicted to be the lowest energy at MP2 level. The energetics between these three stable geometries are calculated to be deviated by a trivial difference at both MP2 and MM levels, as summarized at Table S1 in Supplementary Materials. Two hydrogen-bonded geometries, denoted as NHN and OHO, were selected for comparing the current MEA potential at the MM level with BLYP-D2, as shown in Figure 3. Constrained optimizations were conducted to converge the minimum geometry searching with the XH···X angle frozen at 180°. The intermolecular interactions between two MEA molecules are summarized in Table 2. The binding energy of OHO was predicted to be stronger than that of NHN at −6.90 and −6.15 kcal/mol for the BLYP-D2 and MM levels, respectively. NHN interactions were estimated to be −4.62 and −4.16 kcal/mol for the BLYP-D2 and MM levels, respectively. Even by using the BLYP-D2-optimized geometry, the MM level predicted acceptable interactions compared with the MM-optimized geometry, as shown by ^1^Eint versus ^2^Eint in Table 2. This consistency indicated strong similarity between the two constrained optimized geometries at the BLYP-D2 and MM levels. However, weak contribution to the intermolecular binding from the vdW interactions was still in presence for the current MEA potential. Phase transition of the (MEA)_128_ model was simulated to investigate the thermodynamic property prediction by using the current MEA model. Eight NVT simulations were performed at 280–420 K, with a density of 1.01 g/cm^3^. Each run was pre-equilibrated and sampled for 3 ns to predict the corresponding heat capacity. A phase transition was identified at approximately 320 K as shown in Figure 4, and the experimental freezing point of MEA(l) was 283 K [47]. Thus, the current MEA potential slightly overestimated the melting property of MEA.

### 2.4. CO_2_ Binding Using MEA

**Figure 5:**
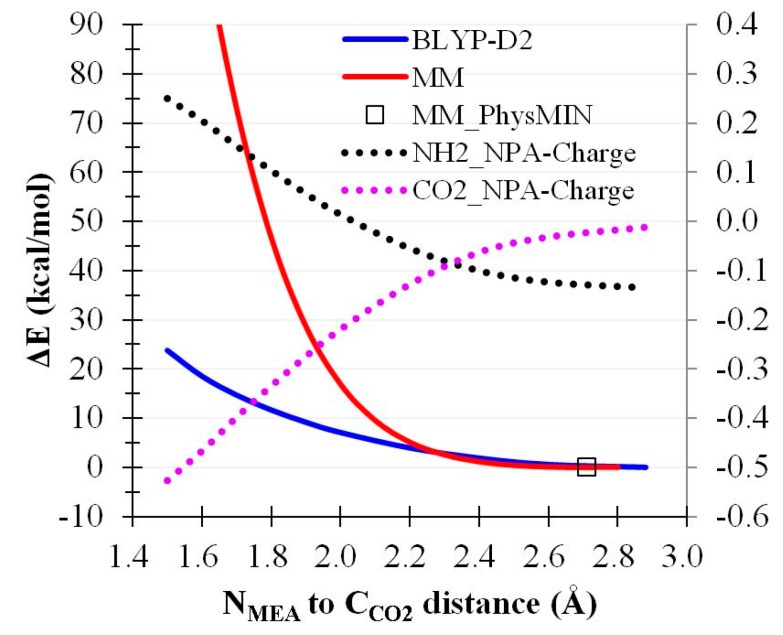
Relative energetics of CO_2_ binding by MEA in the gas phase (kcal/mol).

Constrained optimizations in the gas phase along the C_CO2_–N_MEA_ distance were calculated at both the BLYP-D2 and MM levels along the CO_2_-binding pathway. The relative energetics considering each physical absorption minimum structure at both levels is summarized in Figure 5. CO_2_ was physically absorbed at a distance of approximately 2.9 Å from N_MEA_ at the BLYP-D2/aug-cc-pVTZ level and approximately 2.7 Å for the case predicted at the MM level. Close agreement was observed between the two theory levels for the C_CO2_–N_MEA_ distance (r_CN_) ranging from 2.8 to 2.2 Å. At r_CN_ = 2.0 Å, MM showed more than 2.5 kcal/mol deviated from BLYP-D2. This deviation exponentially increased as r_CN_ increased beyond 2.0 Å. Natural population analysis was performed to estimate the fragment charge of the amino group of MEA (q_NH2_) and CO_2_ (q_CO2_) along the absorption pathway, as plotted at the secondary *y*-axis in Figure 5. At r_CN_ = 2.2–2.8 Å, q_NH2_ and q_CO2_ slightly varied. However, considerable increases and decreases were observed for q_NH2_ and q_CO2_ beyond r_CN_ = 2.0 Å, respectively, suggesting a substantial charge transfer effect as CO_2_ approached the amino group of MEA. The current nonpolarizable potential did not consider such electronic redistribution, regardless of the reparameterization of the balance between electrostatic and vdW interactions in this approach. Thus, marked deviations were anticipated between the MM and BLYP-D2 levels. Nonetheless, the accuracy of the current potential was reasonable at r_CN_ > 2.0 Å.

### 2.5. CO_2_(g) Dissolution by MEA(l)

**Figure 6:**
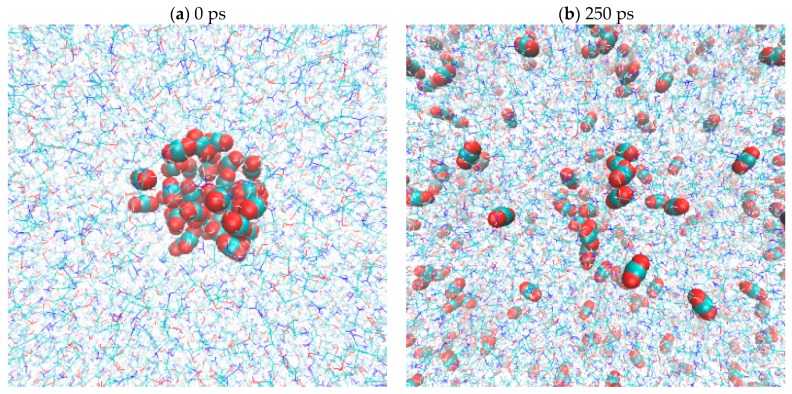
Schematic representation of the initial and final snapshots of CO_2_(g) dissolved in MEA(l).

A conceptual interfacial model using high-density CO_2_(g) surrounded by MEA(l) was characterized to study the solvation dynamics of CO_2_ in MEA(l). CO_2_(g) represented by a (CO_2_)_44_ cluster model was equilibrated at 400 K for 4 ns under NVT ensemble. A repulsive spherical potential (r = 9.1257 Å) was applied, and the sphere density was fixed at 1.01 g/cm^3^. The final atomic coordinates and velocities of the (CO_2_)_44_ cluster were adopted for the subsequent CO_2_(g)/MEA(l) simulation. A 4 ns NVT simulation of (MEA)_864_ under the periodic boundary condition, T = 400 K, and density = 1.01 g/cm^3^ was conducted for creating the MEA(l) model. The MEA molecules located within r = 9.5 Å were removed from the final structure of (MEA)_864_ simulation, and an (MEA)_804_ model was obtained by adopting the corresponding atomic velocities. Finally, the initial condition of the CO_2_(g)/MEA(l) simulation was generated by combining the (CO_2_)_44_ cluster and (MEA)_804_ model by using the same cell size as that of the (MEA)_864_ simulation, as shown in Figure 6.

**Figure 7:**
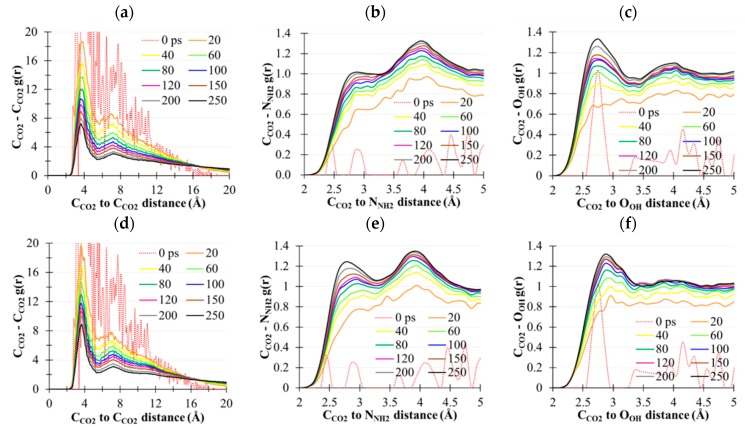
Radial distribution functions of C_CO2_–C_CO2_, C_CO2_–N_NH2_, and C_CO2_–N_OH_ calculated at various time periods. (**a**–**c**) shows the unscaled $\mu_{OH}$ and (**d**–**f**) shows 10% $\mu_{OH}$.

A 250 ps simulation using the isoenthalpic-isobaric ensemble (NPH) was subsequently conducted for the interfacial CO_2_(g)/MEA(l) model. The external pressure of the NPH ensemble was 7415 atm, according to the average value of the previous (MEA)_864_ NVT simulation. Radial distribution functions, g(r), of C_CO2_–C_CO2_, C_CO2_–N_NH2_, and C_CO2_–O_OH_, denoted as $g_{CcCc}^{orig}\left(r\right),\;g_{CcN}^{orig}\left(r\right),\;and\;g_{CcO}^{orig}\left(r\right)$, were plotted considering the simulation time, as shown in Figure 7. Multiple g(r) results were analyzed for various time periods (0, 20, 40, 60, 80, 100, 120, 150, 200, and 250 ps), as shown in the legends. In Figure 7a, $g_{CcCc}\left(r\right)$ substantially increased for the $r_{CcCc}>16$ Å region after 250 ps simulation, and this increase represented the depletion kinetics of high-density CO_2_(g). The profound $g_{CcCc}^{orig}\left(r=3.7\;Å\right)$ indicated the presence of a CO_2_ clustering effect. Such an effect is considered to diminish with the increasing simulation time because of the stronger interaction of CO_2_–MEA than of CO_2_–CO_2_. Between $g_{CcN}^{orig}\left(r\right)$ and $g_{CcO}^{orig}\left(r\right)$, the probability of CO_2_ binding was higher by the hydroxyl group than by amino groups of MEA. Notably, such a difference cannot be attributed to the binding energy of CO_2_ to the amino and hydroxyl groups of MEA because the intermolecular interaction of CO_2_ with the binding motif at the MM level was 5.50 and 5.05 kcal/mol, respectively. According to 250 ps simulation, the hydroxyl groups contributed more to the depletion of the (CO_2_)_44_ cluster and suppression of the interactions between the CO_2_ and amino groups.

**Figure 8:**
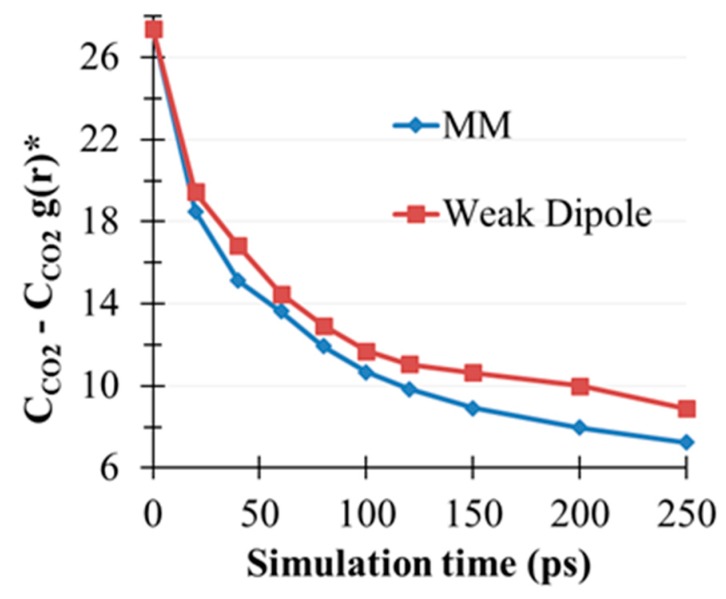
Time evolution for g(r) of C_CO2_-C_CO2_ at $r_{CcCc}=3.65$ Å for the original and scaled simulations.

A control experiment using 250 ps CO_2_(g)/MEA(l) simulation was performed to clarify the role of the hydroxyl groups of MEA, in which $\mu_{OH}$ was scaled down by 90% and other parameters were the same as those in prior unscaled simulation. The resulting g(r) of C_CO2_–C_CO2_, C_CO2_–N_NH2_, and C_CO2_–N_OH_, denoted as $g_{CcCc}^{sc}\left(r\right)$, $g_{CcN}^{sc}\left(r\right)$, and $g_{CcO}^{sc}\left(r\right)$, are summarized in Figure 7d–f. Figure 7d shows that the $g_{CcCc}^{sc}\left(3.7\right)$ peak was substantially higher than $g_{CcCc}^{orig}\left(3.7\right)$ at the end of 250 ps, suggesting the presence of a stronger CO_2_ clustering effect. In other words, CO_2_(g) depletion was suppressed because of weak $\mu_{OH}$••$\mu_{C=O}$ interactions. The physical absorption of CO_2_ by the amino groups was markedly enhanced, as observed in $g_{CcN}^{sc}\left(2.8\right)$ in Figure 7e. The probability of CO_2_ being physically absorbed by the hydroxyl groups was observed at further $r_{CcO}$ distance by $g_{CcO}^{sc}\left(3.0\right)$ compared with $g_{CcO}^{orig}\left(2.7\right)$ in Figure 7c of the unscaled case. The time evolution of $g_{CcCc}^{orig}\left(3.65\right)$ and $g_{CcCc}^{sc}\left(3.65\right)$ was compared with consideration of the simulation time, as shown in Figure 8, and CO_2_(g) depletion was substantially enhanced because of the introduction of the reduced $\mu_{OH}$ of MEA molecules.

The geometries of MEA molecules containing the intramolecular hydrogen bond interactions are analyzed for the 2 ns bulk MEA simulation and the first 200 ps of the CO_2_(g)/MEA(l) simulation. Two hundred snapshots are recorded for each case where 10 MEA molecules are selected and monitored out of these recorded snapshots. The histograms of the intramolecular N_NH2_-H_OH_ distance (r_intraNH_) are recorded in Figure S2 (10 × 200 data points are included at each figure). By comparing the recorded data points of r_intraNH_ < 2.2 Å, the CO_2_(g)/MEA(l) simulation contains substantially more intraHB geometries during the first 200 ps over the case of bulk MEA simulation. The less hydrophilic CO_2_ phase could stabilize the presence of intraHB MEA orientations, while the bulk MEA liquid could favor the intermolecular hydrogen bond interactions.

## 3. Experimental Section

Molecular mechanics (MM) calculations were performed using Tinker version 7.1 [48]. Force field parameters for MEA and CO_2_ adopted the relevant fragments defined in MM3 force field, followed by comprehensive reparameterization routines in order to maximize the agreement with the quantum mechanical (QM) calculations at MP2 or DFT levels for the hydrogen-bonded complexes of (MEA)_2_ and the CO_2_••MEA physically absorbed structure, as discussed below. MM3 was originally designed for describing hydrocarbons and thus provided a good starting point for the subsequent parameterizations [34,35,36,37,38,39]. BLYP functional [49,50] augmented with Grimme’s D2 dispersion correction [51], being reported to appropriately describe CO_2_–alcoholamine interactions by using a triple-zeta quality basis set, was adopted for the DFT calculations [52]. The CO_2_ parameters took into account molecular stretching and bending motions, interatomic Lennard–Jones interactions, and bond-centered dipole–dipole interactions. The interatomic van der Waals (vdW) interactions were described using the arithmetic and geometric combining rules for radius and epsilon, respectively. The cutoff values of vdW and dipole–dipole interactions were set at 10.0 and 12.0 Å, respectively. Berendsen thermostat was used for the molecular dynamic simulations with a coupling time of 0.1 ps [53]. All electronic structure calculations in this study were performed using the GAUSSIAN 09 package [54]. MP2 and BLYP-D2calculations used the aug-cc-pVTZ basis set.

## 4. Conclusions

In the present study, a nonpolarizable MM approach was reported to characterize CO_2_(g) dissolution in MEA(l). The current CO_2_ and MEA potentials showed reasonable agreement with the MP2 and BLYP-D2 levels of theory for describing the intramolecular vibration and intermolecular interactions of CO_2_, (CO_2_)_2_, and (MEA)_2_. Heat capacity simulations by using (CO_2_)_13_ clusters reproduced the solid–solid phase transition at approximately 90 K, as reported in previous studies. The melting transition predicted using the (MEA)_128_ model was approximately 320 K, in which the experimental freezing potential of pure MEA was 283 K. Along the CO_2_ absorption pathway by MEA, the MM approach reproduced the energetics of the BLYP-D2 level if CO_2_ was bound at a distance of approximately 2.0 Å from the amino group of MEA. The absence of polarizable terms in the current potential could not describe the charge transfer effect, which was included in the BLYP-D2 level of theory, because CO_2_ was absorbed by MEA at a short C_CO2_ to N_MEA_ distance (<2.0 Å).

At the gas–liquid interface represented by the (CO_2_)_44_ and (MEA)_804_ models, CO_2_(g) was almost completely depleted within 250 ps. There are notably more intramolecular hydrogen bond interactions identified than the case of bulk MEA simulation. The hydroxyl groups of MEA more actively approached CO_2_ at the interfacial region and suppressed the absorption probability of the NH groups. The artificial reduction of the dipole moment of the hydroxyl bonds enhanced CO_2_–amino group interactions, but the depletion of CO_2_ gas phase was reduced as well. The hydroxyl groups of MEA were identified to play contradictory roles in the interfacial CO_2_ capture process. The current findings could provide additional perspectives for the synthetic design of molecular CO_2_ scrubbers.

## Figures and Tables

**Table 1:** Comparison of CO_2_ monomeric and dimeric models at the BLYP-D2, MP2, and molecular mechanics (MM) levels (kcal/mol).

^1^ Isomers	Min	Parallel	T-Type	$\nu_{\text{CO}2}$ (cm^−1^)
^2^ BE(BLYP-D2)	−1.06	0.25	−0.77	636, 1302, 2310
BE(BLYP)	0.29	0.76	0.09	
BE(disp)	−1.36	−0.50	−0.86	
^2^ BE(MP2)	−1.27	−0.01	−1.07	668, 1310, 2374
BE(HF)	−0.05	0.83	−0.16	
BE(disp)	−1.22	−0.84	−0.91	
^3^ Eint(MM)	−1.23	0.16	−0.92	629, 1240, 2374
${\text{Eint}}_{\mu \mu }^{\text{MM}}$	−1.00	0.32	−0.70	
${\text{Eint}}_{\text{LJ}}^{\text{MM}}$	−0.23	−0.15	−0.22	

^1^ Graphical representation of the (CO_2_)_2_ models is presented in Figure 1. ^2^ Binding energy (BE) (BLYP-D2 and MP2) contains the correction of the basis set superposition error by using BLYP-D2 and MP2 optimized structures, respectively. ^3^ Eint denotes the interaction energy at the MM level, where μμ and LJ denote dipole–dipole and Lennard–Jones terms, respectively.

**Table 2:** Comparison of intermolecular interactions between the BLYP-D2 and MM levels (kcal/mol).

Isomers ^3^	NHN	OHO
^1^ BE(BLYP-D2)	−4.62	−6.90
^1^ BE(BLYP)	−1.35	−3.50
^1^ BE(disp)	−3.28	−3.32
^1^ ${\text{Eint}}_{\mu \mu }^{\text{MM}}$	−1.85	−3.61
^1^ ${\text{Eint}}_{\text{VDM}}^{\text{MM}}$	−1.88	−1.40
^2^ ${\text{Eint}}_{\text{Total}}^{\text{MM}}$	−4.16	−6.15
^2^ ${\text{Eint}}_{\mu \mu }^{\text{MM}}$	−2.17	−4.33
^2^ ${\text{Eint}}_{\text{VDM}}^{\text{MM}}$	−1.99	−1.82

^1^ Constrained optimized geometry at the BLYP-D2/aug-cc-pVTZ level. ^2^ Constrained optimized geometry at the MM level. ^3^ The same notation is adopted as that in Table 1.

## Supplementary Materials

The following are available online at: http://www.mdpi.com/1420-3049/22/1/8/s1. optimized geometries of MEA monomer at MP2 and MM levels; histograms of potential interHB geometries in the MD simulations; Cartesian coordinates at BLYP-D2/aug-cc-pVTZ level for the OHO and NHN; force field parameters for CO_2_ + MEA
