# Supplementary material for: Solvation Dynamics of CO2(g) by Monoethanolamine at the Gas–Liquid Interface: A Molecular Mechanics Approach
Source: Molecules. 2016 Dec 23;22(1):8. doi: 10.3390/molecules22010008 (PMC6155642; doi:10.3390/molecules22010008)
Supplement: Supplementary file 1 [file molecules-22-00008-s001.pdf]

Supplementary Materials: Solvation Dynamics of CO_2(g) by Monoethanolamine at the Gas–Liquid Interface: A Molecular Mechanics Approach

I-Shou Huang, Jia-Jen Li and Ming-Kang Tsai

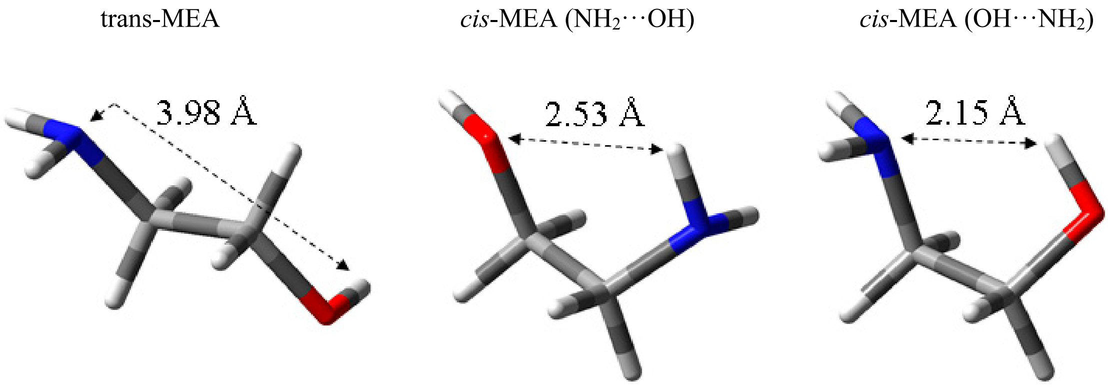

Figure S1. MP2/aug-cc-pVTZ optimized monomeric MEA geometries using PCM model in EtOH. The distance between N of NH_2 group to H of OH group is labeled.

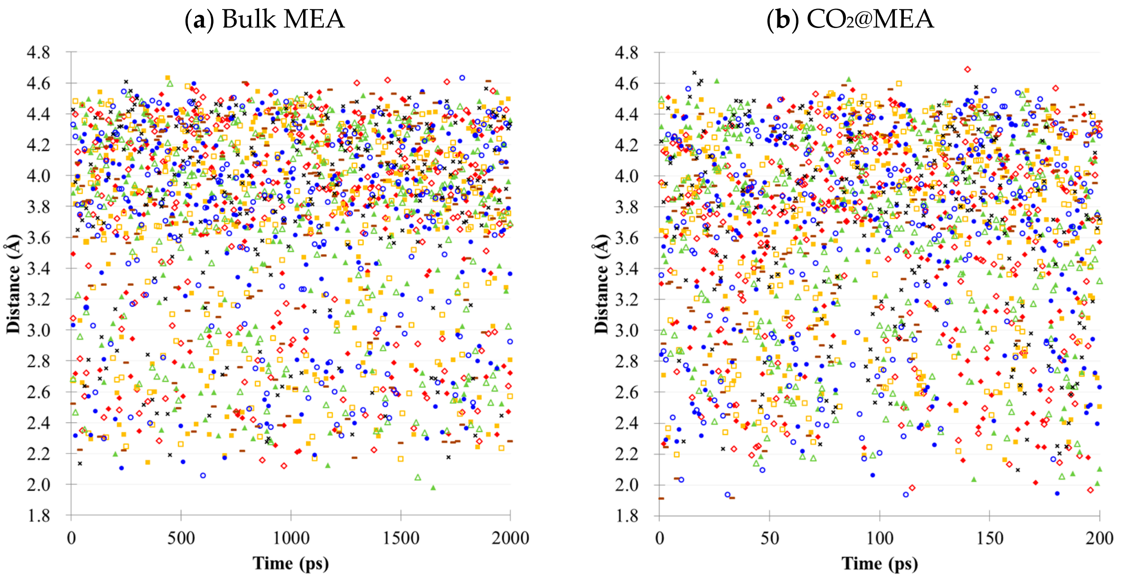

Figure S2. History of intramolecular N_{NH2}-H_{OH} distance in Å of (**a**) 2 ns $(MEA)_{128}$ bulk simulation at 400 K and (**b**) $(CO_2)_{44}$ in $(MEA)_{804}$ simulations at 400 K. The sampled potential energy surface of simulation (**a**) is shown in Figure S3. Ten MEA molecules are randomly selected and N-H distance is recorded per 10 ps for (**a**). The 10 MEA molecules positioning closest to $(CO_2)_{44}$ at t = 0 ps are chosen and N-H distance is recorded per 1 ps for (**b**).

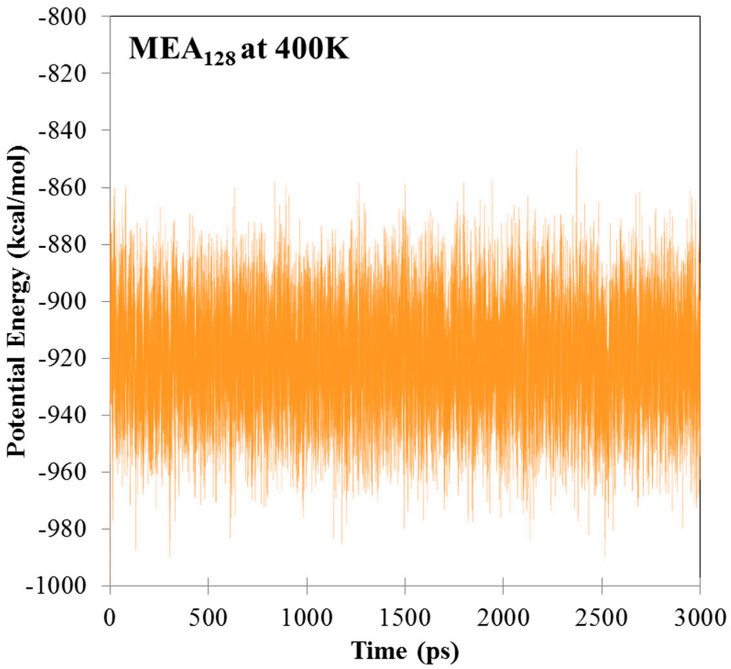

Figure S3. The 3 ns history of the sampled potential energy for bulk MEA at 400 K and the last 2 ns trajectory is used for intramolecular HB analysis.

Table S1. The energetics of various stable geometries calculated at MP2/aug-cc-pVTZ using PCM model and MM level in vacuum.

kcal/mol (AU)	E_{PCM}(MP2)	G_{PCM}(MP2)	ΔU(MM)
trans-MEA	0.00 (−210.02476)	0.00 (−209.95389)	0.00
cis-MEA (NH_2···OH)	−1.01 (−210.02636)	−0.91 (−209.95534)	−0.02
cis-MEA (OH···NH_2)	−2.55 (−210.02881)	−1.83 (−209.95682)	0.18

Table S2. Cartesian coordinates at BLYP-D2/aug-cc-pVTZ level for the OHO and NHN.

OHO				NHN			
N	3.023	−0.080	−1.310	N	−1.655	1.716	0.176
H	2.112	−0.453	−1.585	H	−1.684	2.590	−0.348
H	3.719	−0.524	−1.910	H	−1.883	1.944	1.146
C	3.263	−0.433	0.107	C	−2.645	0.753	−0.369
H	4.225	−0.001	0.419	C	−2.431	−0.613	0.288
H	3.304	−1.523	0.291	H	−2.473	0.661	−1.449
C	2.156	0.153	0.985	H	−3.695	1.059	−0.219
H	2.397	−0.028	2.045	H	−1.435	−0.997	0.027
H	2.090	1.237	0.811	H	−2.489	−0.504	1.387
O	0.893	−0.480	0.674	O	−3.478	−1.493	−0.187
H	0.249	0.221	0.436	H	−3.227	−2.402	0.038
O	−0.970	1.548	−0.015	H	0.165	0.525	−0.201
H	−1.265	1.978	0.806	N	1.010	−0.028	−0.375
N	−3.638	−1.087	−0.274	H	1.420	0.303	−1.250
H	−3.265	−1.653	−1.038	C	1.982	0.188	0.713
H	−4.065	−1.735	0.388	C	3.270	−0.580	0.445
C	−2.542	−0.335	0.379	H	1.548	−0.190	1.651
H	−1.664	−0.946	0.646	H	2.244	1.252	0.872
H	−2.940	0.103	1.306	H	3.941	−0.508	1.318
C	−2.095	0.797	−0.558	H	3.032	−1.637	0.249
H	−1.729	0.385	−1.506	O	3.903	0.016	−0.722
H	−2.939	1.468	−0.772	H	4.603	−0.584	−1.024

parameter set for CO_2 + MEA parameterized by MKT (2016)#

atom	1	C	“CSP3 ALKANE”	6	12.000	4	
atom	5	H	“EXCEPT ON N,O,S”	1	1.008	1	
atom	3	C	“C of CO2”	6	12.000	2	
atom	7	O	“O of CO2”	8	15.999	1	
atom	6	O	“C-O-H, C-O-C, O-O”	8	15.995	2	
atom	21	H	“-OH ALCOHOL”	1	1.008	1	
atom	8	N	“NSP3”	7	14.003	3	
atom	23	H	“NH AMINE/IMINE”	1	1.008	1	
bond	1	5	4.86	1.0969			
bond	6	21	7.57	0.9718			
bond	8	23	6.42	1.0222			
bond	1	8	5.30	1.4811			
bond	1	6	5.70	1.4478			
bond	1	1	4.49	1.5297			
bond	3	7	14.50	1.1739			
angle	7	3	7	0.70	180.00		
angle	5	1	5	0.51	109.44		
angle	21	6	21	0.66	104.56		
angle	23	8	23	0.45	106.78		
angle	5	1	8	0.82	109.30		
angle	1	8	23	0.60	110.20		
angle	1	6	21	0.75	108.00		
angle	1	1	5	0.59	109.80		
angle	1	1	6	0.83	107.50		
angle	1	1	8	0.78	109.47		
angle	5	1	6	0.82	110.00		
torsion	5	1	1	8	0.000 0.0 1	0.000 180.0 2	0.374 0.0 3
torsion	6	1	1	8	0.000 0.0 1	−1.050 180.0 2	1.850 0.0 3
torsion	5	1	1	5	0.000 0.0 1	0.000 180.0 2	0.238 0.0 3
torsion	5	1	1	6	0.000 0.0 1	0.000 180.0 2	0.300 0.0 3
torsion	1	1	6	21	0.400 0.0 1	0.000 180.0 2	0.100 0.0 3
torsion	5	1	6	21	0.000 0.0 1	0.000 180.0 2	0.200 0.0 3
torsion	1	1	8	23	0.073 0.0 1	−0.422 180.0 2	0.327 0.0 3
torsion	5	1	8	23	0.121 0.0 1	−0.648 180.0 2	0.199 0.0 3
vdw	6	1.820	0.059				
vdw	8	1.930	0.043				
vdw	1	2.040	0.800				
vdw	3	1.412	0.030				
vdw	7	1.513	0.100				
dipole	1	8	1.65	0.5			
dipole	1	6	1.94	0.5			
dipole	3	7	1.80	0.5			
dipole	6	21	−1.94	0.5			
dipole	8	23	−1.65	0.5			
